# Clozapine-Associated Neutropenia in a Middle Eastern Population: Incidence, Predictors, and the Role of Benign Ethnic Neutropenia

**DOI:** 10.1192/bjo.2026.11945

**Published:** 2026-06-30

**Authors:** Syed Ali Bokhari, Muhanad Elnoor, Faisal Nawaz, Syed Fahad Javaid

**Affiliations:** 1 Al Amal Psychiatric Hospital, Emirates Health Services, Dubai, UAE; 2 Department of Psychiatry, College of Medicine and Health Sciences, United Arab Emirates University, Al Ain, UAE

## Abstract

**Aims:**

Clozapine-associated neutropenia occurs in approximately 3.8% of patients globally, with agranulocytosis reported in around 0.9%. Benign ethnic neutropenia (BEN), which is prevalent among Arab and African populations, complicates interpretation of haematological monitoring and may lead to unnecessary treatment interruption. Despite the high representation of BEN-susceptible ethnicities in Gulf populations, regional data on clozapine haematological safety are lacking. This study aimed to estimate the incidence of clozapine-associated neutropenia, identify clinical predictors, and evaluate the role of baseline absolute neutrophil count (ANC) in risk stratification.

**Methods:**

A retrospective cohort study was conducted including all patients prescribed clozapine at a tertiary psychiatric hospital in the United Arab Emirates between January 2018 and July 2024. A total of 184 patients were followed over 228.1 patient-years, comprising 3,907 ANC measurements. Neutropenia was defined as ANC <2.0 × 10^9^/L and categorised as mild (1.5–2.0), moderate (1.0–1.5), severe (0.5–1.0), or agranulocytosis (<0.5). Cox proportional hazards regression was used to identify predictors of neutropenia. Receiver operating characteristic (ROC) analysis assessed the discriminatory performance of baseline ANC.

**Results:**

Twenty patients (10.9%; 95% CI 7.1–16.2%) developed neutropenia, corresponding to an incidence rate of 9.74 per 100 patient-years (95% CI 5.95–15.05). Most events were mild (n=19, 10.3%), and no cases of agranulocytosis were observed. Patients of Arab or African ethnicity had significantly higher neutropenia rates compared with other ethnic groups (16.2% vs 1.6%; odds ratio 11.5, 95% CI 1.5–87.5; p=0.001). Baseline ANC emerged as the only independent predictor of neutropenia (hazard ratio 0.15, 95% CI 0.07–0.31; p<0.001), with each unit increase associated with an 85% reduction in risk. Baseline ANC demonstrated excellent discriminatory ability (AUC 0.92, 95% CI 0.86–0.97), with an optimal threshold of 3.3 × 10^9^/L (sensitivity 85%, specificity 87.4%). Clozapine was continued in 80% of affected patients, with no permanent discontinuations due to haematological events.

**Conclusion:**

This first Middle Eastern study of clozapine haematological safety demonstrates neutropenia rates comparable to international data and no observed agranulocytosis. Baseline ANC provides excellent risk stratification, while higher neutropenia rates in Arab and African patients likely reflect benign ethnic neutropenia rather than true drug toxicity. These findings support continued clozapine treatment with appropriate monitoring and highlight the need for ethnicity-informed interpretation of haematological results in diverse populations.

No financial sponsorship was received for this project.

